# Decreased Task-Related HRV Is Associated With Inhibitory Dysfunction Through Functional Inter-Region Connectivity of PFC in Major Depressive Disorder

**DOI:** 10.3389/fpsyt.2019.00989

**Published:** 2020-01-22

**Authors:** Hongliang Zhou, Zongpeng Dai, Lingling Hua, Haiteng Jiang, Shui Tian, Yinglin Han, Pinhua Lin, Haofei Wang, Qing Lu, Zhjjian Yao

**Affiliations:** ^1^ Department of Psychiatry, the Affiliated Brain Hospital of Nanjing Medical University, Nanjing, China; ^2^ School of Biological Sciences & Medical Engineering, Southeast University, Nanjing, China; ^3^ Child Development and Learning Science, Key Laboratory of Ministry of Education, Nanjing, China; ^4^ Nanjing Brain Hospital, Medical School of Nanjing University, Nanjing, China

**Keywords:** major depressive disorders, magnetoencephalography, task-related heart rate variability, functional connectivity, go/no-go task

## Abstract

The regulation of the autonomic nervous system (ANS) can improve cognitive function in major depressive disorders (MDD). Heart rate variability (HRV) derives from the dynamic control of the ANS and reflects the balance between the activities of the sympathetic and parasympathetic nervous systems by measuring tiny changes in adjacent heart beats. Task-related HRV may reflect the association between the flexibility of cognition and ANS function. The study was to investigate the neural mechanism of interactions between ANS and cognitive function in MDD with Magnetoencephalography (MEG) measurements. Participants included 20 MDD patients and 18 healthy controls (HCs). All participants were measured with a go/no-go task MEG. HRV indices, the standard deviation of the average normal-to-normal (NN) interval calculated over short periods (SDANN) and the square root of the mean squared differences of successive NN intervals (RMSSD), were derived from the raw MEG data. Results showed that MDD patients showed decreased SDANN and RMSSD. In MDD patients, both resting-state and task-related RMSSD were related to inhibitory and control dysfunction. In the go/no-go task, many areas in the prefrontal cortex (PFC) are responsible for an individual’s inhibitory function. A brain MEG functional connectivity analysis revealed that there were significant differences in four brain regions within the prefrontal cortex (PFC) between MDD patients and HCs. Task-related RMSSD in HCs were related to the functional connectivity between the left middle frontal gyrus and the anterior cingulate cortex (ACC), while in MDD patients, these values were not related to the above functional connectivity but were related to the functional connectivity between the left middle frontal gyrus and insula. However, the resting-state RMSSD value was not related to these significant difference functional connectivity networks in all participants. It concludes that the decreased task-related HRV is associated with inhibitory dysfunction through functional inter-region connectivity in the PFC in MDD, and the task-related HRV can be used as an index of the association between MDD and autonomic dysregulation.

## Introduction

Major depressive disorder (MDD) is a serious health care problem worldwide, according to the report of illness-induced disability (1). MDD is a more severe state of depressive symptomatology in which patients present multiple depressive symptoms and show significant distress or impaired functioning. Of the many depressive symptoms, cognitive impairments are common in MDD. Cognitive function is an individual’s ability to process information. Cognitive dysfunction is prevalent and is associated with the early onset of depression and with longer depressive episodes in MDD patients; it also has an adverse impact on treatment outcomes, as well as on functional recovery (2). Inhibitory and control function is a critical cognitive function that can be defined as interference control, prepotent response inhibition, and resistance to interference. Prepotent response inhibition is the ability to suppress or withhold a (motor) response, which can be measured by the go/no-go task (3).

Inhibitory and control function includes two levels: a central level, which is related to the cerebral cortex, and a peripheral level, which is mediated by the autonomic nervous system (ANS) (4). At the central level, many studies using neuroimaging methods have reported that in the go/no-go task, many areas in the prefrontal cortex (PFC), such as BA9, BA10, and BA47, are responsible for an individual’s inhibitory function (5–9). At the peripheral level, the ANS, especially the vagus nerve, plays an important role in an individual’s inhibitory and control function (4). Many studies have shown that the decreased vagus tone is associated with abnormal inhibitory and control function under the emotional stop-signal task or the Stroop test (10, 11).

Magnetoencephalography (MEG) has high temporal and spatial resolution and has been used to investigate the neural mechanism of many cognitive functions (12). Functional connectivity measures how brain regions are temporally coordinated and is employed to probe brain network architecture (13). MEG has been used for investigations of brain cortical activity and functional connectivity in MDD (14). MEG in MDD patients allows inter-regional and intraregional connectivity interactions in brain function to be related to cognition, which can provide mechanistic insights into interactions between the ANS and cognitive function.

Individual ANS functioning relies on a balance between the activities of the sympathetic and parasympathetic nervous systems. Although cardiac automaticity is originated in pacemaker tissues, heart rate and rhythm largely depend on the control of ANS (15). By releasing neurotransmitters such as acetylcholine, epinephrine, and norepinephrine, the sympathetic and parasympathetic nervous systems can affect heart beats which lead to the changes of heart rate variability (HRV). HRV derives from the dynamic control of the ANS and reflects the balance between the activities of the sympathetic and parasympathetic nervous systems by measuring tiny changes in adjacent heart beats. The measurement of HRV contains time domain methods, frequency domain methods and rhythm pattern analysis. Time domain methods and frequency domain methods are mostly applied to clinical researches. Although frequency domain methods are computed by decomposing waveform of electrocardiogram (ECG)–RR intervals (interbeat interval or RRIs) and are considered to better distinguish the influence of the parasympathetic and sympathetic nervous systems, they may be affected by different therapeutic regimes and other factors easily comparing with time domain methods which may also reflect the activities of the sympathetic and parasympathetic nervous systems (16–18). The standard deviation of the average normal-to-normal (NN) intervals (SDANN), which is calculated over a short period (usually within 5 min), and the square root of the mean squared differences of successive normal sinus intervals (RMSSD) are components of HRV from the time-domain analysis. SDANN represents sympathetic function, and RMSSD represents the flexibility of vagal (parasympathetic) tone (19, 20). It is accepted that HRV is decreased in MDD (21–23), and decreased HRV might be associated with abnormal perseveration cognition (24). A recent study proved that yoga could be an add-on treatment for MDD by adjusting HRV (25). In addition, HRV biofeedback (HRVB), which has been extensively applied in the clinic, can improve cognition and other symptoms of MDD (26–30). It is worth noting that according to the different experimental conditions, HRV can be measured as the resting-state HRV or task-related HRV. Previous studies that used resting-state HRV investigated the association between the ANS and cognition in MDD, and confirmed that resting-state HRV can be used as a characteristic biological marker for MD (23, 24, 31).

Because task-related HRV may reflect the association between the flexibility of cognition and ANS function, task-related HRV can be used as a sensitive index to reflect inhibitory control function at the peripheral level (32). However, to date, no studies on the association between the ANS and cognition (i.e. inhibitory control function) in MDD, which was shown to be involved in task-related HRV, have been reported. Most importantly, the neural mechanism of interactions between the ANS and inhibitory and control function in MDD remains unclear.

In this study, both resting-state HRV and task-related HRV were used to assess ANS function; a go/no-go task was employed to measure cognitive function (inhibitory and control function), and MEG was used to measure brain cortical activity and functional connectivity. To guarantee cognition along different psychophysiological dimensions, task-related HRV indices were derived from the raw MEG data. The purpose of this study is to 1) uncover the neural mechanism of interactions between the ANS and inhibitory and control function in MDD and 2) confirm whether task-related HRV can be used as an index of the association between MDD and autonomic dysregulation.

## Materials and Methods

### Time and Setting

The experiment was completed in the Department of Psychiatry, Affiliated Nanjing Brain Hospital of Nanjing Medical University, Nanjing, People’s Republic of China, from January 1, 2017, to July 28, 2019. All experimental procedures were approved by the Ethics Committee on Human Studies, Affiliated Nanjing Brain Hospital of Nanjing Medical University, Nanjing, People’s Republic of China and were conducted depending on the Declaration of Helsinki (Ethical review number: 2016KY12). All patients and healthy controls signed an informed consent form, and they received 20 United States dollars as a reward after finishing the experiment. Considering that some patients could not sign the consent form, legal guardians who could represent these patients were also asked to provide consent for all experimental procedures.

### Diagnostic Approaches and Participants

This study included an MDD group and an HC group. The criteria for the MDD group were as follows: 1) only meet the criteria of Diagnostic and Statistical Manual of Mental Disorders, Fifth edition (DSM-5) for major depression; 2) age from 18 years old to 65 years old; 3) Hamilton Depression Scale (17-item edition, HAMD) scores ≥ 17; 4) no fewer than two episodes of depression; 5) had not taken medication for the last two weeks; 6) were not treated with electroconvulsive therapy within the last month; 7) no diagnosis of alcohol, drug, or other substance dependency, any kind of head injury, neurological disorder, or systemic disease that might have an effect on the central nervous system; and 8) no contraindications for the MEG measurements. The criteria for the HC group were as follows: 1) did not meet the criteria of any DSM-5 axis I disorder or personality disorders according to the Structured Clinical Interview for DSM-5 (SCID-5, Chinese version); 2) age from 18 years old to 65 years old; 3) HAMD (17-item edition) scores ≤ 7; 4) no history of any kind of psychiatric disorder; 5) no diagnosis of alcohol, drug, or other substance dependence; and 6) no diagnosis of any kind of head injury, neurological disorder or systemic disease that might have an effect on the central nervous system. All participants needed to be free of any form of cardiac disease, lung disease, beta-blocker medication, or any other disease that could influence the physiological data, and they were forbidden to drink any coffee or tea on the day of the test session. In addition, to reduce the stress caused by MEG examination, patients were informed the whole process of the research and were asked to fit the environment in advance after 8 min resting.

According to the above criteria, twenty patients with MDD were chosen from thirty-four inpatients, and eighteen healthy persons were chosen from thirty healthy volunteers. These HCs were citizens who lived in Nanjing city, Jiangsu Province, People’s Republic of China. All participants were Chinese.

On the day of the experiment, with the assistance of a psychiatric resident physician, a psychiatric associate chief physician was asked to collect medication information, demographic and clinical characteristics, and confirm/exclude the MDD diagnosis of participants. We used the Annett Handedness Scale to evaluate handedness. The standards of handedness were as follows: Annett score (1) = right, (2–7) = mixed, and (8) = left. All participants were right handed.

### MEG Task and Procedure

#### Go/No-Go Task

**Figure 1:**
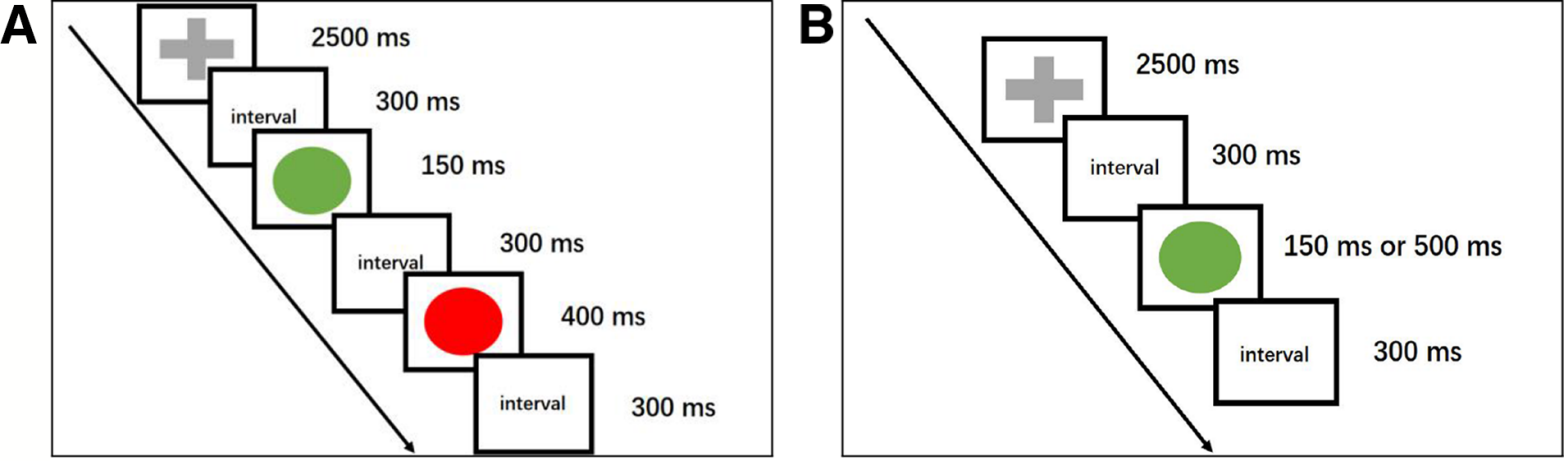
A cartoon illustrating the go/no-go task. ms, millisecond. **(A)** This schematic representation of a no-go trial begins with the presentation of a gray fixation cross (go stimulus), followed by a green lamp (go stimulus). Then, a red lamp, which indicates a no-go trial, is shown, and participants need to respond. The no-go trials accounted for 25% of the entire session, and every picture was followed by a 300 ms interval. **(B)** The go trials consisted of a gray fixation cross whose duration was 2,500 ms and a green lamp. The duration of the green lamp was randomly either 150 or 500 ms.

The go/no-go task was edited by BrainX software, which was based on DirectX (Microsoft Corporation, Redmond, WA, USA). One hundred eighty stimuli were used in this task, and the total duration of the task was 300 s. Three types of stimuli were used in this task: a red lamp picture, a green lamp picture, and a gray cross. These stimuli were shown serially on a white background against a black background (1.5 × 1.5 cm in size) on a computer screen. The whole task contained both go and no-go trials. In a go trial, the participant was shown a gray cross on a white background that was presented for 2,500 millisecond (ms) followed by a green lamp picture. The green lamp picture remained on the screen for either 150 or 500 ms, decided at random. In a no-go trial, a red lamp picture was presented for 400 ms duration, followed by a gray cross picture and then a green lamp picture. The duration of the gray fixation cross presentation was 2,500 ms and that of the green lamp was 150 ms. After every picture was presented, there was an interval time of 300 ms. The duration of no-go trials accounts for 25% of the entire task. There were 20 go trials and 5 no-go trials in this experiment in the practice phase, and the data in the practice phase were not recorded or analyzed (**Figure 1**).

### Behavioral Data Measurements

The behavioral data included the accuracy rate (hit rate, i.e. the percentage of correct responses), reaction times (RTs), and false alarms (error rate, i.e. the opposite of the intended action was selected on accident). The accuracy rate and RTs were recorded for go trials, and false alarms were recorded for no-go trials.

### MEG Data Acquisition

A 275-channel whole-head CTF MEG system (Omega 2000, VSM Med Tech Inc., Port Coquitlam, Canada) was used for the MEG recordings. The sampling rate of the machine was adjusted to 1,200 Hz. Any metal on participants’ bodies was removed, and people were asked to lay in the supine position in a special magnetically shielded room. With a third-order synthetic gradient, the system could cancel background and noise interference effectively. During MEG measurements, the movement of head motion and localization could be tolerated within 5 millimeter (mm). The T1-weighted imaging data were obtained from Siemens Verio 3.0 Tesla Magnetic Resonance Imaging (MRI) scanner (Erlangen, Germany). The T1-weighted axial image parameters were as follows: repetition time/echo time (TR/TE) = 1,900/2.48 ms, thickness/gap = 1.0/0 mm, field of view (FOV) = 240 × 240 mm^2^, matrix = 256 × 256 × 192, and voxel size = 1 × 1 × 1 mm^3^. Three energizing coils were set at the nasion, left preauricular, and right preauricular locations to measure the participant’s head location within the scanner and for offline co-registration of MRI and MEG data.

#### Pre-Processing

The Fieldtrip toolbox (http://www.ru.nl/fcdonders/fieldtrip/) in a MATLAB software environment (http://www.mathworks.com) was used to pre-process MEG data. After removing the frequency band between 49.5 and 50.5 Hz, which indicated big noise that could affect the authenticity of the data, the raw MEG data were separated into three parts: go epochs, no-go epochs and interval epochs. Only correct no-go epochs were used in the MEG analysis. The time range of 200 ms pre-stimulus and 900 ms post-stimulus during no-go epochs was selected. The SPM megheadloc function was applied to screen the data for head motion by removing any epochs with motion greater than 5 mm or when intertrial movement was >10 mm. Then, we used independent component analysis (ICA) to eliminate eye movement and cardiac artifacts. The 1- to 120-Hz frequency band was chosen for analysis.

#### Source Reconstruction and Functional Connectivity Calculations

The minimum-norm estimates algorithm was used to complete source reconstruction after pre-processing the raw MEG data (33). We used averaged envelope correlation (AEC) to compute the functional connectivity networks within brain regions between MDD patients and HCs under the go/no-go task. The filtered time series gotten after source reconstruction were transformed by Hilbert transform to get the analytic signal, and then the absolute value of the signal was extracted and divided into several parts. The time consistency between each two parts of them was calculated by average correlation value with Pearson correlation analysis. Considering that there had been no similar study carried out before, according to previous studies (34, 35), we chose the prefrontal lobe, which is the area primarily reactive to the go/no-go task. Significantly, the anterior cingulate cortex was specifically chosen for its tight association with HRV. Although there are some doubts surrounding exploring deep brain areas by MEG, considering that the important role of the insula plays in HRV, this region was chosen as an auxiliary brain area for the explanation of our results. The brain areas chosen to compute function connectivity in the no-go epoch are shown in **Table 1**.

**Table 1:** Brain regions chosen from previous studies.

Area	H	X	Y	Z	BA
Superior frontal gyrus	L	−24	48	22	9
	L	−26	62	8	10
	L	−48	28	−16	47
	R	26	46	42	8
	R	42	60	2	10
	R	24	54	34	8
	R	30	60	−14	10
	R	22	62	24	9
Middle frontal gyrus	L	−28	56	−2	10
	L	−30	48	36	8
	R	−50	48	18	46
	R	38	38	30	9
	R	22	36	42	8
Insula	L	−35.13	6.65	3.44	13
	L	−32	16	−16	13
	L	−34	12	−14	13
	R	44	−6	−4	
Anterior cingulate cortex		4	26	−4	

### HRV Data Acquisition and Analysis

To guarantee HRV could reflect task-related state more precisely, ECG record was directly collected from the MEG raw data under the go/no-go task after ICA (36). There were two reasons to choose time domain analysis. Firstly, the bandpass frequency range of MEG data was set from 0 HZ to 120 HZ, which could disturb the indices of frequency domain analysis. Secondly, time domain analysis is not sensitive to the usage of medication other than tricyclic and tetracyclic antidepressants (37), which could minimize the influence of medication. By recording continuous ECG data, each QRS complex was detected with the pan-tompkin function in the MATLAB software environment, and the normal-to-normal (NN) intervals which were all intervals between adjacent QRS complexes resulting from instantaneous heart rate were determined. And HRV components SDANN and RMSSD were selected to be analyzed. The pan-tompkin function in the MATLAB software environment was applied to lock the R wave. Then, the RMSSD and SDANN were measured with the following formulas: SDANN (which represents the activity of sympathetic nervous system) $=\sqrt{\frac{1}{\text{N}}\sum_{i-1}^{\text{N}}{\left(\text{R}{\text{R}}_{i}-\bar{\text{R}}\bar{\text{R}}\right)}^{2}}$ and RMSSD (which represents the activity of parasympathetic nervous system) $=\sqrt{\frac{1}{\text{N}-1}\sum_{i=1}^{\text{N}-1}{\left(\text{R}{\text{R}}_{i+1}-\text{R}{\text{R}}_{i}\right)}^{2}}$ (38). Resting-state HRV was derived from the recording data of HRV biofeedback software after 8 min resting at the same time (about 2 o’clock in the afternoon) on the next day to reduce the effect of circadian rhythm on experiment. Although the data contain all indices of HRV, to match task-related HRV, only the SDANN and RMSSD values were used for analysis.

### Data and Statistical Analysis

Data are shown as the means (standard deviation, SD). Statistical Program for Social Sciences software version 19.0 (SPSS, IBM Corporation, Armonk, NY, USA) was applied to statistical analysis. Independent-samples *t*-tests and paired-sample *t*-tests were applied to compare mean age, education, The Hamilton Depression Scale (HAMD, 17-item edition) scores, the Hamilton Anxiety Scale (HAMA) scores, behavioral data, HRV values, and functional connectivity of brain regions between the MDD and the HC groups, and the Pearson chi-square test was used to investigate the gender ratio. A general linear correlation method was used to investigate the relationship between HRV indices and significant difference functional connectivity networks, and HAMD scores used Pearson’s *r* in the MDD patients and the HCs. To reduce the likelihood of false positives, the false-discovery rate (FDR) correction was used to correct multiple comparisons (39). Alpha values of 0.05 were considered to have significance.

## Results

### Demographic Characteristics of Participants

As shown in **Table 2**. There were no significant differences between the demographic characteristics of the participants in the MDD group and the HC group. The HAMA scores were higher in the MDD group than in the HC group; however, there were no significant differences. The HAMD scores of the MDD group were higher than those of the HC group.

**Table 2:** Demographic and clinical characteristics of participants.

	MDD	HC	Test statistic
Gender ratio (M/F)	20 (8/12)	18 (7/11)	*χ* ^2^ = 0.005, *p* = 0.944
Mean age (SD)	31.6 (9.8)	30.7 (8.2)	*t* = 0.291, *p* = 0.773
Age range	19–48	19–44	—
Age of onset (SD)	27.9 (6.04)	—	—
Education (SD)	12.6 (2.3)	14.1 (2.8)	*t* = 1.89, *p* = 0.67
Total duration of depressive episode (month, SD)	39.6 (10.7)	—	—
Number of depressive episode onset (SD)	3.7 (1.1)	—	—
HAMA (SD)	7.8 (2.1)	5.0 (3.3)	*t* = 1.985, *p* = 0.060
HAMD (SD)	24.9 (6.3)	5 (1.4)	*t* = 13.749, *p* = 0.000

MDD: major depressive disorders; HC, healthy control; F, female, M, male; SD, standard deviation; R, right, M, mixed, L, left;. HAMA, Hamilton Anxiety Scale; HAMD, Hamilton Depression Scale.

### Behavioral Data Analysis

As shown in **Table 3**, RTs for go trials in the MDD group were longer than those in the HC group; the hit rate for go trials in the MDD group was lower than that in the HC group, and the rate of false alarms for no-go trials in the MDD group was higher than that in the HC group.

**Table 3:** Comparisons of behavioral data between MDD and HC group [present as mean (SD)].

Group	MDD (n = 20)	HC (n = 18)	*t*	*p*
RTs (go trials, ms)	470.5 (26.1)	385.8 (12.9)	9.701	0.000
Hit rate (go trials)	0.823 (0.040)	0.916 (0.025)	7.187	0.000
False alarms (no-go trials)	0.209 (0.050)	0.102 (0.112)	3.641	0.000

MDD, major depressive disorder group; HC, healthy control group; ms, millisecond.

### HRV Indices Analysis

#### Comparisons of HRV Indices Between Different States

**Figure 2:**
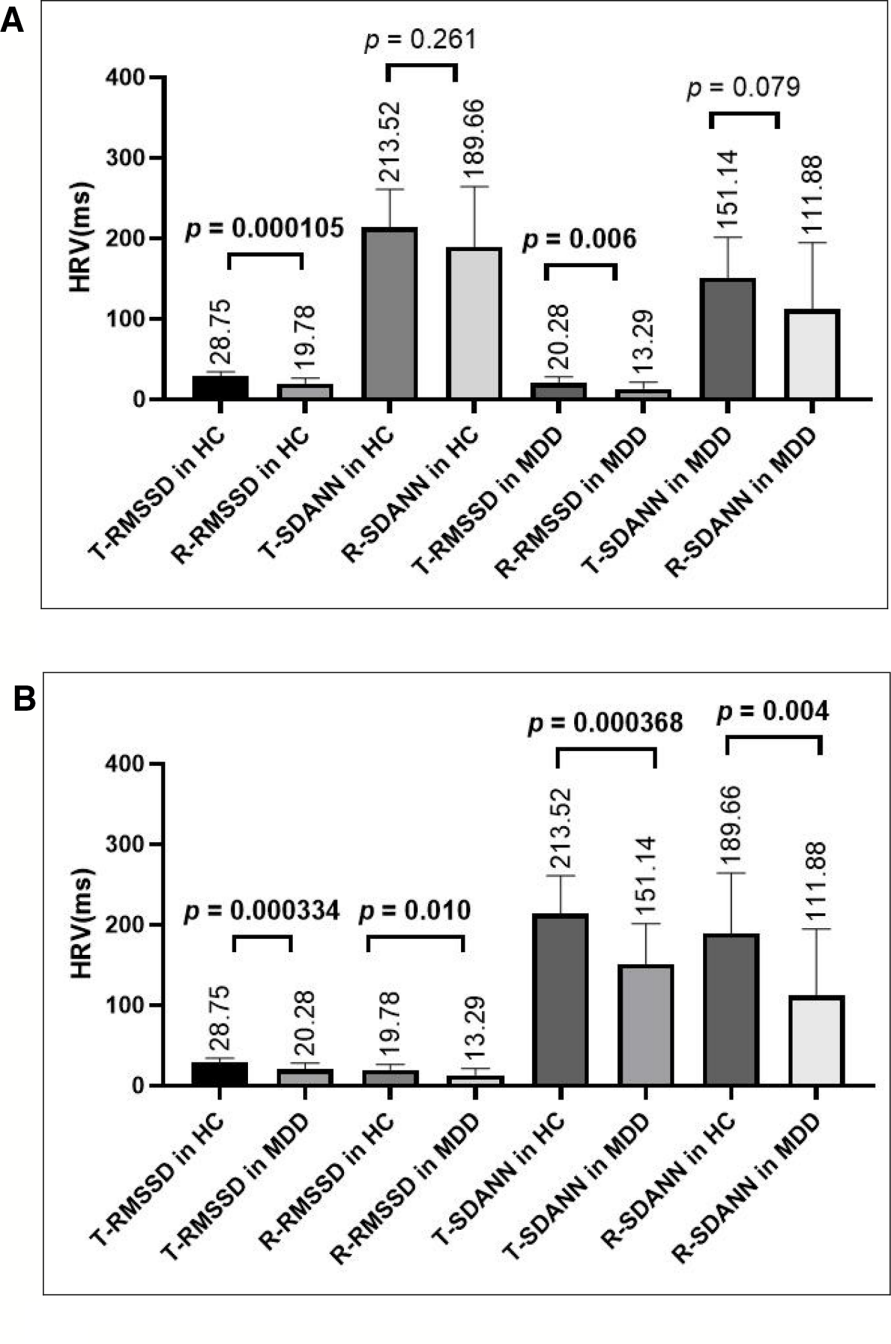
**(A)** Comparing HRV indices in the task-related state or resting state within the HC group or MDD group, there was no difference in the SDANN value, but there was a significant difference in the RMSSD value. **(B)** Comparing HRV indices in the resting state or the task-related state between the HC and MDD group, there were significant differences in both the RMSSD and SDANN values. T-RMSSD, task-related RMSSD, R-RMSSD, resting-state RMSSD; T-SDANN, task-related SDANN, R-SDANN, resting-state SDANN.

As shown in **Figure 2A**, by paired-sample *t*-test, there were no differences in SDANN values in the resting state and task-related state (in the Go/No-go task) in the HC group or in the MDD group (for HC group, *t* = 1.661,*p* = 0.115; for MDD group, *t* = 1.856, *p* = 0.079). However, there were significant differences in the RMSSD value in both the HC group and the MDD group (for the HC group, *t* = 5.667, *p* = 0.000028; for the MDD group, *t* = 2.993, *p* = 0.007); the RMSSD value in both the HC group and the MDD group in the resting state were lower than that in the task-related state.

#### Comparisons of HRV Indices Between the HC Group and the MDD Group

As shown in **Figure 2B**, by independent sample *t-*test, there were significant differences in both the SDANN and RMSSD values in the resting state (for SDANN, *t* = 3.034, *p* = 0.004; for RMSSD, *t* = 2.712, *p* = 0.010) and in the task-related state (for SDANN, *t* = 3.932, *p* = 0.000368; for RMSSD, *t* = 3.965, *p* = 0.000334) between HCs and MDD patients. Both SDANN and RMSSD values in MDD patients were lower than those in HCs in both the resting state and the task-related state.

#### Correlation Analysis Between HRV Indices and Behavioral Data

The general linear correlation method was used to investigate the relationship among HRV indices (resting-state SDANN and RMSSD values and task-related SDANN and RMSSD values) and behavioral data (RTs, hit rate, and false alarms). The results showed that only the resting-state RMSSD value and the task-related RMSSD value were correlated with RTs in the MDD group (for resting-state RMSSD: *r* = −0.476, *p* = 0.034; for task-related RMSSD: *r* = −0.522, *p* = 0.018).

### MEG Analysis

**Figure 3:**
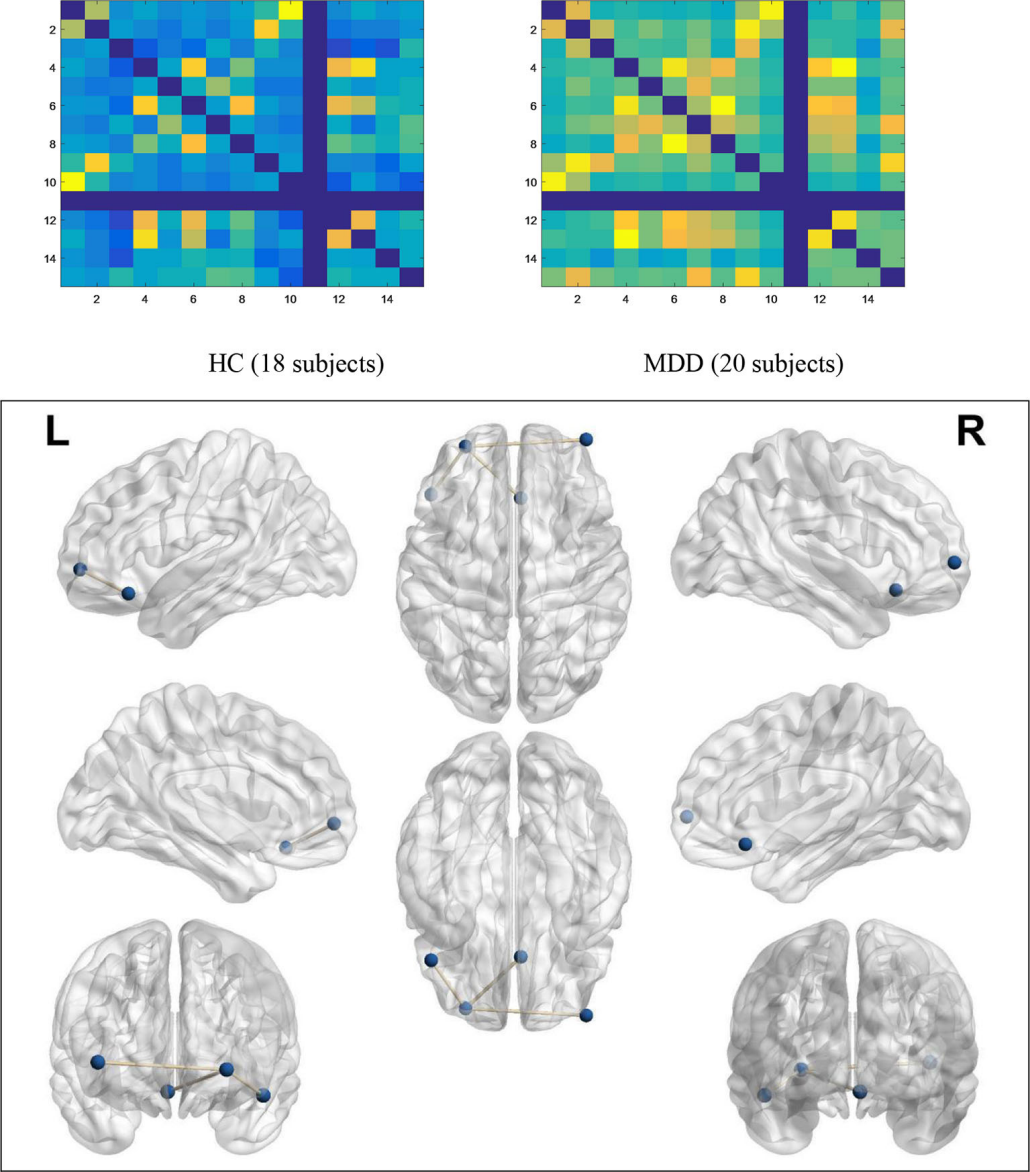
Functional connectivity networks of significant differences. In the no-go trials, the average activity of functional connectivity networks within PFC in the MDD group (20 subjects) was higher than that in the HC group (18 subjects), which means that MDD patients have to activate more connections than HCs to control their behavior.

As shown in **Figure 3**, compared to the brain regions in the HC group, there were three regional functional connectivities in the brain that passed FDR correction in the MDD group, including the functional connectivity between the left middle frontal gyrus (−28, 56, −2) and right superior frontal gyrus (42, 60, 2) (*t* = −3.573, *p* = 0.001027), the functional connectivity between the left middle frontal gyrus (−28, 56, −2) and left superior frontal gyrus (−48, 28, −16) (*t* = 3.972, *p* = 0.000375), and the functional connectivity between the left middle frontal gyrus (−28, 56, −2) and anterior cingulate cortex (4, 26, −14) (*t* = −3.851, *p* = 0.000569).

### Correlation Analysis Between the RMSSD Value and Significant Difference Functional Connectivity Networks

#### Task-Related RMSSD Value

A general linear correlation method was used to investigate the relationship between task-related HRV indices and the three above mentioned brain regional functional connectivity networks; this analysis showed that in the HC group, the task-related RMSSD value is positively correlated with the functional connectivity between left middle frontal gyrus (−28, 56, −2) and anterior cingulate cortex (4, 26, −14) (*r* = 0.605, *p* = 0.008), while in the MDD group, the task-related RMSSD value is not correlated with above functionally connected regions (*r* = −0.117, *p* = 0.625), and, instead, the task-related RMSSD value is negatively correlated with the functional connectivity between left middle frontal gyrus (−28, 56, −2) and insula (44, −6, −4) (*r* = −0.577, *p* = 0.008). However, we didn’t find correlation between task-related RMSSD and the functional connectivity between left middle frontal gyrus (−28, 56, −2) and left insula (−35.13, 6.65, 3.44), (−32, 16, −16), (−34, 12, −14) (*r* = −0.17, −0.197, −0.115; *p* = 0.306, 0.235, 0.317).

#### Resting-State RMSSD Value

A general linear correlation method was used to investigate the relationship between the resting-state RMSSD value and the three above mentioned regional functional connectivity networks in the brain; this analysis showed that the RMSSD value was not correlated with these significant difference brain regions in either the HC group or the MDD group (for HC group: *r* = 0.225, 0.173, 0.166; *p* = 0.369, 0.493, 0.511; for MDD group: *r* = −0.205, −0.008, −0.99; *p* = 0.495, 0.855, 0.532).

### Correlation Analysis Between the HAMD Value and Significant Difference Functional Connectivity Networks or HRV Indices in the MDD Group

By Pearson correlation analysis, the HAMD scores in the MDD group were positively correlated with the functional connectivity between the left middle frontal gyrus (−28, 56, −2) and right superior frontal gyrus (42, 60, 2) (*r* = 0.516, *p* = 0.020); the HAMD scores in the MDD group were negatively correlated with HRV indices (for task-related RMSSD: *r* = −0.555, *p* = 0.011; for task-related SDANN: *r* = −0.559, *p* = 0.010; for resting-state RMSSD: *r* = −0.497, *p* = 0.026).

## Discussion

This study is the first to employ brain MEG functional connectivity analysis with a go/no-go task, by combining HRV indices, to uncover the neural mechanism of interactions between the ANS and inhibitory and control function in MDD patients. Additionally, this study is the first to confirm that task-related HRV can be used as an index of the association between MDD and autonomic dysregulation.

ANS, which is generally regarded made of sympathetic system and parasympathetic system, is one of the most important factors that are associated with a wide range of somatic and mental disease. Although pacemaker tissues play an important role in cardiac automaticity, heart rate, and rhythm largely depended on the neurotransmitters (acetylcholine, epinephrine, and norepinephrine) secreted by sympathetic nervous system and parasympathetic nervous system. HRV, which represent the rhythm of heart, has been used as an indicator of central–peripheral neural feedback (40). SDANN and RMSSD are two important indices in HRV which reflect sympathetic nervous system and parasympathetic nervous system respectively. There are growing studies to discover the relationship between ANS and central nervous system (CNS) based on neurovisceral integration model, and investigators have identified functional units within the CNS (such as PFC) which are related to several cognitive functions such as executive function, memory, attention, etc. (41). Notably, PFC also plays an important role in inhibition and control function, and there are several researches to find vagally mediated HRV is associated with inhibition and control function.

Consistent with previous studies (22, 42–44), MDD patients present inhibitory and control dysfunction and decreased resting-state RMSSD values. Compared to the task-related state, all participants displayed lower resting-state HRV values. Additionally, MDD patients showed lower HRV indices (SDANN and RMSSD values) than HCs in both the resting state and the task-related state. Furthermore, in MDD patients, both the resting-state and the task-related RMSSD value were related to inhibitory and control dysfunction. A brain MEG functional connectivity analysis in MDD patients revealed that four brain regions within the PFC had significant difference functional connectivity networks. Meanwhile, task-related RMSSD values in HCs were related to the functional connectivity between the left middle frontal gyrus and the anterior cingulate cortex (ACC), while in MDD patients, this measure was not related to the above mentioned functionally connected regions but was instead related to the functional connectivity between the left middle frontal gyrus and insula. However, the resting-state RMSSD value was not related to significant difference functional connectivity networks in all participants.

Our results also support that resting-state HRV can be used as a biomarker of inhibitory and control function in MDD patients. A previous study indicated that task-related HRV may reflect inhibitory and control function at the peripheral level (32). Our results support the theory that the decreased vagus nerve function might be the mechanism of the decreased HRV indices in MDD patients. In addition, comparing HRV indices in the resting state with those during the task (the go/no-go task), the RMSSD value was closely related to the vagus function and, thus, might be a better marker to index impaired inhibitory and control function than that of the SDANN value, which is associated with the sympathetic nervous system in MDD patients. Our results also confirm that the task-related HRV may reflect the association between the flexibility of cognition and ANS function.

The fundamentals of the PFC function are the “inhibition” of the ordinary and stereotyped responses in order to pursue the novelty “execution”. Although PFC plays an important role in so many functions (such as inhibitory, work memory, regulation of visceral feeling, etc.), in this study, the function of inhibitory was highlighted. The PFC plays a critical role in inhibitory and control function, which can be projected to BA8, 9, 10, 11, 12, 13, 44, 45, 46, and 47 Brodmann areas (7, 8). The left middle frontal gyrus (BA10) and right superior frontal gyrus (BA47), seen in this study, belong to the PFC. It is widely recognized that the PFC plays an important role in the regulation of HRV (44–47). A previous study has shown that the middle frontal gyrus and superior frontal gyrus are sensitive brain regions related to inhibitory and control function (48–51). In addition, a study reported that the right superior frontal gyrus is one of the targets of erythropoietin treatment, which can effectively improve the executive function of MDD patients (52). As an important part of the limbic system, the anterior cingulate cortex (ACC) is not only associated with the regulation of inhibitory and control function but also with the regulation of breathing and heart rate. The reduction in dopamine D2 receptors in the ACC is an important pathological mechanism of MDD, and increasing ACC dopamine D2 receptor levels can effectively improve executive function in MDD patients (53). In the go/no-go task, abnormal regional functional connectivity networks in the brain are the neural mechanism of cognitive dysfunction (inhibitory control dysfunction) in MDD. The appearance of “visceral brain” components suggest that decreased HRV indices resulting from the sympathetic-vagus nerve balance disorder are associated with inhibitory and control dysfunction.

Unlike in HCs, HRV indices are not associated with the functional connectivity between the left middle frontal gyrus and anterior cingulate cortex in MDD patients. We suggest that MDD patients have abnormal activation in regional brain functional connectivity, decreasing the regulation of sympathetic-vagus nerve function by the ACC. Namely, this abnormal activation decreases the regulation HRV indices by the ACC; however, to compensate for the decreased regulation of HRV indices by the ACC, as the regulation of the vital center of the body, the insula strengthens the regulation of sympathetic-vagus nerve function. In addition, the functional connectivity networks between insula (no matter in right hemisphere or left hemisphere) and PFC were higher in MDD patients than that in HCs, which supported our idea that the functional connectivity between insula and PFC could compensate for abnormal functional connectivity networks within PFC. However, it is worth noting that, we only took insula into consideration as an auxiliary brain region to explain our results in that there was still controversy about discovering the function about deep brain regions with MEG.

## Limitations

There are some limitations to this study. Firstly, because of the small sample size, our results are preliminary. Secondly, although MEG data integrated with task-related HRV indices has verified that task-related HRV is a characteristic biological marker of MDD in this study, whether task-related HRV can be used as a genuine diagnostic indicator for MDD depends on multidimensional research. Future studies should integrate MEG with other technologies, such as genetic and immunohistochemical techniques, to further confirm the relationship between task-related HRV indices and MDD. Finally, our study only investigated the neural mechanism of interactions between ANS and inhibitory and control function in MDD with MEG measurements. Because inhibitory and control function was only one of neurocognitive functions, other aspects of neurocognitive such as working memory, sustained attention, etc. should be conducted in the future study.

## Conclusions

In conclusion, the decreased task-related HRV is associated with inhibitory control dysfunction through functional inter-region connectivity in the PFC in major depressive disorder, and the task-related HRV be used as an index of the association between MDD and autonomic dysregulation.

Our findings have important implications for understanding the pathophysiology of MDD and task-related HRV could be used to predict abnormal inhibitory and control function in MDD patients. In addition, integrated with task-related HRV, MEG may provide an image-guided tool for intervention.

## Data Availability Statement

The raw data supporting the conclusions of this manuscript will be made available by the authors, without undue reservation, to any qualified researcher.

## Ethics Statement

The studies involving human participants were reviewed and approved by the Ethics Committee on Human Studies, Affiliated Nanjing Brain Hospital of Nanjing Medical University, Nanjing, People’s Republic of China. The patients/participants provided their written informed consent to participate in this study.

## Author Contributions

HZ: data curation, formal analysis, methodology, writing original draft, writing review and editing. LH, HJ, ZD, ST, YH, PL, HW: data curation, formal analysis, methodology. QL, ZY: data curation, formal analysis, methodology, writing review and editing.

## Funding

This work was supported by the National Natural Science Foundation of China [grant number 81871066 and 81571639]; the Jiangsu Provincial Medical Innovation Team of the Project of Invigorating Health Care Through Science, Technology and Education [grant number CXTDC2016004]; and the Jiangsu Provincial Key Research and Development Program [grant number BE2018609]. The funders had no role in study design, data collection and interpretation, or the decision to submit the work for publication.

## Conflict of Interest

The authors declare that the research was conducted in the absence of any commercial or financial relationships that could be construed as a potential conflict of interest.
